# Efficiency and Quantitative Structure-Activity Relationship of Monoaromatics Oxidation by Quinone-Activated Persulfate

**DOI:** 10.3389/fchem.2021.580643

**Published:** 2021-09-01

**Authors:** Jiaqi Shi, Tao Long, Yuxuan Zhou, Lei Wang, Cuiping Jiang, Dongguo Pan, Xin Zhu

**Affiliations:** ^1^State Environmental Protection Key Laboratory of Soil Environmental Management and Pollution Control, Nanjing Institute of Environmental Sciences, Ministry of Ecology and Environment, Nanjing, China; ^2^College of Environment, Hohai University, Nanjing, China

**Keywords:** persulfate, quinone, degradation kinetics, quantitative structure-activity relationship, monoaromatics

## Abstract

Quinones and quinone-containing organics have potential of activating persulfate to produce sulfate radical. In this work, the optimal condition for quinone activation of persulfate was investigated. It was found representative monoaromatics were degraded fastest in alkaline environment (pH 10.0), but excessive alkalinity restrained the reaction instead. The mechanisms to explain this phenomenon were speculated. The effect of initial quinone concentration on persulfate oxidation was also investigated at pH 10.0. In addition, a quantitative structure-activity relationship model was established with 15 kinds of monoaromatics, which revealed the most negative atomic net charges on carbon atom played an important role on degradation rates. Chemicals with a smaller $q_{\text{C}}^{-}$ were easier oxidized in quinone-activate system. This finding helps further exploration of effective activator in alkaline environment.

## Introduction

Persulfate-based oxidation process is widely used in water and soil remediation, in which persulfate is activated to produce sulfate radical anion (${\text{SO}}_{4}\cdot^{-}$) as the main active material to degrade recalcitrant organics (Waldemer et al., 2007; Xian et al., 2020). The common activation methods include thermal energy, transition metal, alkali and UV radiation, etc. Recently, new activating methods which utilize naturally occurring materials such as metal ores and organics have attracted much attention (Lin et al., 2019; Wu et al., 2019; Zhang et al., 2020). Quinones and organic matters containing quinone-like groups are ubiquitous in natural environment (Siqueira et al., 1991; Cory and McKnight, 2005), and they have been found to be effective as the accelerator or activator in advanced oxidation process (Zhou et al., 2015; Zhang et al., 2019). For instance, quinones increased the oxidation rate of contaminants in Fenton-like system, which was ascribed to their role as an electron shuttle (Duesterberg and Waite, 2007). The presence of quinone contributed to build up semiquinone/quinone and Fe(III)/Fe(II) cycles (Ma et al., 2005). Zhu et al. (2002, 2007) observed the production of hydroxyl radical (OH•) by halogenated quinones and hydrogen peroxide. Fang et al. (2013) found that ${\text{SO}}_{4}\cdot^{-}$ was generated from persulfate in the presence of 1,4-benzoquinone (BQ), 2-methyl-1,4-benzoquinone (MBQ), or 2-chloro-1,4-benzoquinone (MCQ). These reactions were all semiquinone radical dependent. Zhou et al. (2015) revealed that 1,4-benzoquinone could efficiently activate peroxymonosulfate for the degradation of sulfamethoxazole, and the catalytic mechanism was that a dioxirane intermediate formed and was decomposed into ^1^O_2_ subsequently.

Despite those advantages, there are still some unsolved confusions, including the most suitable target chemicals and the optimal working condition of quinone-activated persulfate system. As a result, the influences of pH and quinone concentration on the degradation efficiency of representative volatile organic compound were investigated in this study, and a quantitative structure-activity relationship (QSAR) model for the degradation rate constant (*k*) of monoaromatics was established. This comprehensive work would further reveal the quinone activation mechanisms and provide data support for the application of this oxidation method.

## Methodology

### Chemicals

Sodium persulfate (Na_2_S_2_O_8_), target contaminants (listed in Table 1), sodium thiosulfate (Na_2_S_2_O_3_), sulfuric acid (H_2_SO_4_), sodium hydroxide (NaOH), sodium hydrogen phosphate (Na_2_HPO_4_), sodium dihydrogen phosphate (NaH_2_PO_4_), and sodium borate (Na_2_B_4_O_7_·10H_2_O) were obtained from Sinopharm Chemical Reagent Co., Ltd. The BQ, MBQ, 2,5-dimethyl-1,4-benzoquinone (DMBQ) and the spin-trapping agent 5,5-dimethyl-1-pyrroline-N-oxide (DMPO) were purchased from Aladdin Chemistry Co., Ltd. All reagents were of high purity analytic grade. Solutions in this study were prepared with ultrapure water purified by Millipore deionized water purification system.

**Table 1:** EPR determination condition.

Microwave frequency	9.77 GHz
Microwave power	19.92 mW
Modulation frequency	100 kHz
Modulation amplitude	1.00 G
Time constant	40.96 ms
Sweep time	83.87 s

### Degradation Procedures

All degradation experiments were performed at 20°C in 40 mL glass vial reactors with Teflon inner membrane lids. The concentration of Na_2_S_2_O_8_ and monoaromatics were 5 mM and 100 μg/L, respectively, and the total volume was 40 mL. Samples were withdrawn and quenched with Na_2_S_2_O_3_ periodically before analysis (Huber et al., 2005; Zhou et al., 2015; Zhang et al., 2019). A preliminary study demonstrated that Na_2_S_2_O_3_ did not react with typical organics (1,2-dichloroethane, bromobenzene, and 1,2,4-trichlorobenzene) and had no interference with the analysis of the organics. All the experiments were conducted in triplicate.

In order to explore the optimal conditions of the reaction, different initial pH (pH 3, 7, 10, and 12) and quinone concentrations (0.01, 0.05, 0.1, 0.5, and 1 mM) were set respectively to investigate their effects on the degradation efficiency.

The reaction rate constants used to build the QSAR model were obtained from experiments under the optimal conditions acquired in the above investigation.

### Analysis

The concentration of the monoaromatic was measured by purge & trap autosampler (Eclipse 4552&4660) and GC-MS system (Agilent 7890A/5975C) equipped with a capillary column (DB-624 60 m × 0.25mm × 1.4μm). The column temperature program on GC-MS was set as follows: the beginning temperature of the column oven was 40°C, held for 5 min, increased up to 200°C with a heating rate of 8°C/min and increased up to 230°C with a heating rate of 10°C/min, remained for 2 min. The injector temperature was 250°C and the split ratio was 50:1. The carrier gas was helium.

Electron paramagnetic resonance (EPR) is a spectral method for detecting unpaired electron and can be used to identify free radicals during the degradation process by the technique of spin trapping, which is conducted to produce spin adduct with the diamagnetic compound named spin trapping agent (Johnson et al., 1992; Kevan, 1997). EPR determination in this work was performed as follows: 450 μL samples were withdrawn and mixed with 50 μL DMPO (1 M), and then the solution was injected into a capillary tube, which was inserted into the EPR spectrometer (Bruker, EMX10/12) with a resonance frequency of 9.77 GHz, microwave power of 19.97 mW, modulation frequency of 100 kHz, modulation amplitude of 1.0 G, time constant of 40.96 ms, and sweep time of 83.87 s.

### Model Derivation and Validation

The QSAR model helps understand the oxidation mechanism of quinone-activated persulfate based on the relation between degradation efficiency and molecular structure property. In this work, the structural descriptors (shown in Table 2) came from the manuscript by Zhu et al. (2016), in which the structure optimization and frequency calculation were conducted at B3LYP/6-31^+^G(d,p) level using density functional theory (DFT) method with Gaussian 09. The molecular descriptors included energy of the highest occupied molecular orbital (*E*_HOMO_), energy of the lowest unoccupied molecular orbital (*E*_LUMO_), the largest negative partial net charge on a carbon atom ($q_{\text{C}}^{-}$), the largest positive net charge on a hydrogen atom ($q_{\text{H}}^{+}$), dipole moment (*μ*), total energy (*TE*), surface area (*SA*), molecular volume (*V*m), hydration energy (*HE*), refractivity (*R*), partition coefficient (log *P*) and polarizability (*P*). The total data set (*n* = 20) was divided into training set (*n* = 15) and test set (*n* = 5) at intervals. The chemicals were ordered according to their structures. The ones with the number of 4*n* (*n* = 1, 2, 3, 4, and 5) were classified as the test set for external validation, and the rest were set as the training set to derive the model. The test set chemicals were marked with asterisk (^*^) in Table 2.

**Table 2:** The molecular descriptors of 20 kinds of monoaromatics.

**No**.	**Compound**	***E*_**HOMO**_**	***E*_**LUMO**_**	$q_{\text{C}}^{-}$	$q_{\text{H}}^{+}$	***μ***	***TE***	***SA***	***V*m**	***HE***	***R***	**log*P***	***P***
		**(eV)**	**(eV)**	**(e)**	**(e)**	**(debye)**		**(Å** ^**2**^ **)**	**(Å** ^**3**^ **)**				**(10** ^**−30**^ **esu)**
1	benzene	−6.719	0.073	−0.084	0.084	0.000	−232.3	237.4	329.9	−2.110	26.06	2.050	10.43
2	toluene	−6.411	0.081	−0.381	0.122	0.344	−271.6	268.4	384.2	−1.390	31.10	2.510	12.27
3	ethylbenzene	−6.372	0.098	−0.328	0.112	0.459	−310.9	290.3	432.7	0.190	35.70	2.910	14.10
4^*^	n-propylbenzene	−6.408	0.123	−0.317	0.103	0.325	−350.2	323.7	490.0	1.710	40.30	3.310	15.94
5	isopropylbenzene	−6.425	0.098	−0.304	0.107	0.284	−350.2	320.8	480.8	1.770	40.25	3.240	15.94
6	n-butylbenzene	−6.397	0.125	−0.318	0.103	0.361	−389.5	360.3	545.7	3.190	44.90	3.700	17.77
7	tert-butylbenzene	−6.412	0.101	−0.310	0.105	0.325	−389.5	332.3	518.8	1.870	44.72	3.670	17.77
8^*^	para-xylene	−6.142	0.173	−0.381	0.120	0.096	−310.9	297.3	436.9	−0.720	36.14	2.980	14.10
9	ortho-xylene	−6.248	0.195	−0.381	0.118	0.585	−310.9	286.3	427.8	−0.770	36.14	2.980	14.10
10	1,3,5-trimethylbenzene	−6.148	0.154	−0.381	0.120	0.088	−350.2	322.6	489.8	−0.120	41.18	3.450	15.94
11	1,2,4-trimethylbenzene	−6.014	0.203	−0.382	0.119	0.290	−350.2	315.0	480.6	−0.150	41.18	3.450	15.94
12^*^	para-isopropyltoluene	−6.156	0.100	−0.381	0.121	0.092	−389.5	349.0	533.5	2.400	45.29	3.710	17.77
13	chlorobenzene	−6.714	−0.365	−0.093	0.110	1.918	−691.9	264.5	376.1	−2.900	30.86	2.560	12.36
14	bromobenzene	−6.590	−0.366	−0.086	0.109	1.810	−2803.4	274.7	394.7	−2.890	33.68	2.840	13.06
15	para-chlorotoluene	−6.455	−0.333	−0.382	0.126	2.350	−731.2	292.6	429.4	−2.220	35.90	3.030	14.20
16^*^	ortho-chlorotoluene	−6.568	−0.265	−0.378	0.136	1.665	−731.2	285.2	422.3	−2.270	35.90	3.030	14.20
17	1,4-dichlorobenzene	−6.750	−0.764	−0.094	0.120	0.000	−1151.4	288.2	421.1	−3.720	35.67	3.080	14.29
18	1,2-dichlorobenzene	−6.848	−0.673	−0.088	0.118	2.761	−1151.4	285.2	416.4	−3.760	35.67	3.080	14.29
19	1,2,4-trichlorobenzene	−6.926	−1.037	−0.093	0.139	1.401	−1611.0	310.0	460.7	−4.630	40.47	3.600	16.22
20^*^	styrene	−6.047	−0.853	−0.252	0.101	0.187	−309.7	283.5	416.0	−3.730	35.74	2.700	13.91

* *Test set chemicals*.

The reaction kinetics were explored and the rate constants of 20 kinds of monoaromatics were calculated. The multiple stepwise linear regression (MSLR) analytical method was carried out by SPSS version 17.0 to build the QSAR model.

The quality of derived QSAR was evaluated with the squared regression coefficient (*R*^2^), the standard error of the estimate (*SEE*) and the Fisher's test value (*F*). The model internal predictivity, which reflected the stability of model, was measured by the leave-one-out cross validation coefficient ($Q_{\text{LOO}}^{2}$) and the root mean square of the cross-validation (*RMSE*_cv_). The external validation capability was measured by external validation coefficient ($R_{\text{pred}}^{2}$) and root mean square error of prediction (*RMSE*_p_). It is generally believed that *RMSE*_cv_ < 1 and $Q_{\text{LOO}}^{2}$ > 0.7 indicates the model has a great internal prediction ability, and that $R_{\text{pred}}^{2}$ >0.4 and *RMSE*_p_ < 1 indicates the model has a great external prediction ability (Gramatica, 2007; Puzyn et al., 2008).

## Results and Discussion

### Effect of pH

It has been reported that pH value had a significant effect on persulfate oxidation (Liu et al., 2014; Zhou et al., 2018). We chose an acid (pH 3), a neutral (pH 7), a basic (pH 10.0) and a strongly alkaline environment (pH 12) to investigate the effect of pH on 1,4-benzoquinone-activated persulfate oxidation efficiency. A total of 10 kinds of volatile organic compounds, including 4 kinds of monoaromatics (benzene, *p*-xylene, chlorobenzene, and *p*-chlorotoluene), 3 kinds of chloroalkanes (chloroform, 1,2-dichloroethane, and 1,2-dichloropropane), and 3 kinds of chloroalkenes (trans-1,2-dichloroethylene, trans-1,3-dichloropropylene, and trichloroethylene) were chosen as the target contaminants to investigate the effects of pH. The initial concentrations of Na_2_S_2_O_8_, 1,4-benzoquinone and target contaminant were 5 mM, 0.1 mM, and 100 μg/L, respectively. The pH of acid solution was adjusted with sulfuric acid, and reactions in neutral or alkaline environment occurred in the 10 mM phosphate or borate buffer solutions which had negligible influences on persulfate oxidation efficiency (Fang and Shang, 2012; Fang et al., 2017). The sample were taken and analyzed at 0, 10 min, 30 min, 1 h, 1.5 h, 2 h, 3 h, and 4 h, respectively to observe the variation of concentration with time. Controls at different pH with and without persulfate were set up respectively.

**Figure 1:**
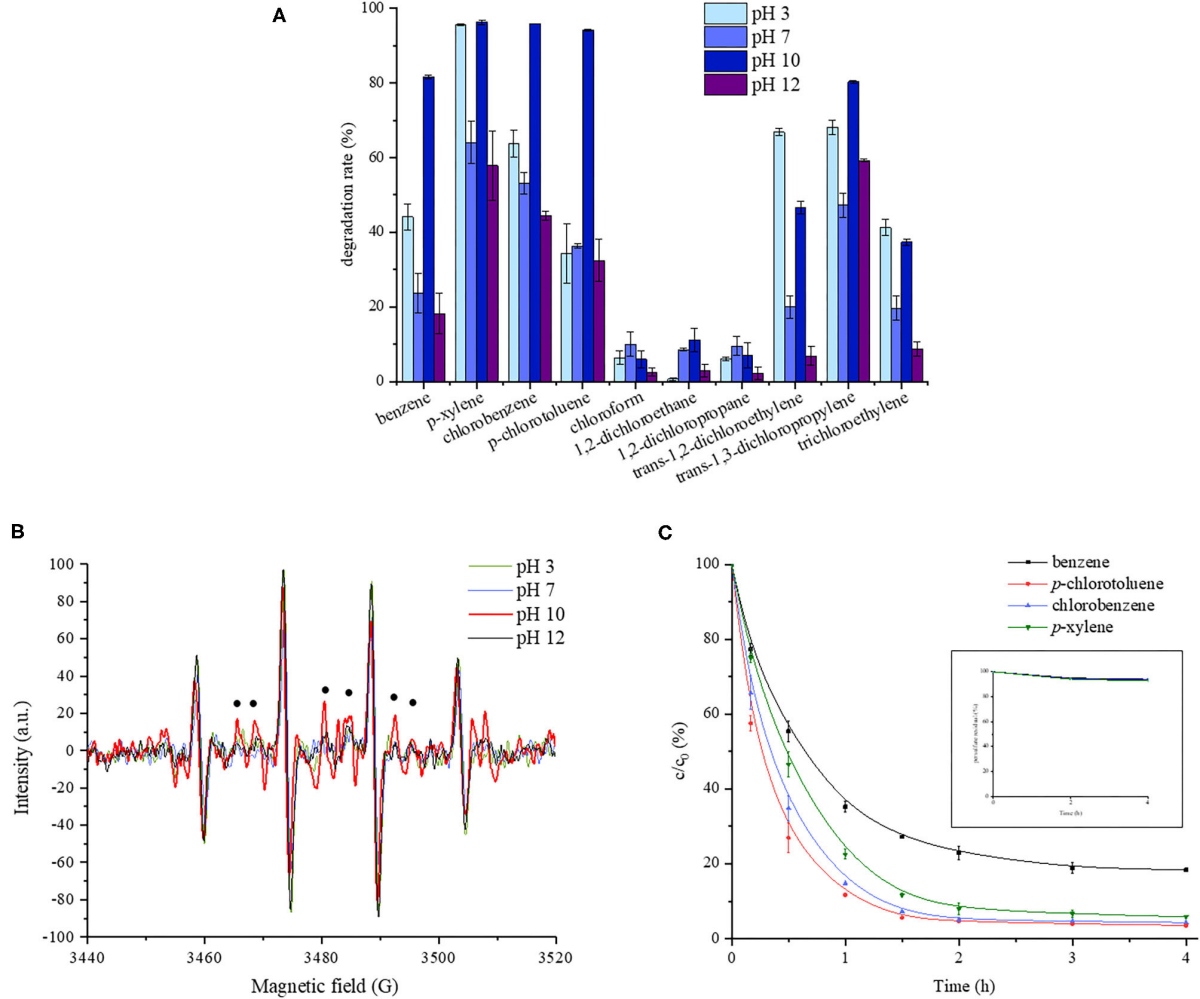
Effect of pH on quinone-activated persulfate oxidation. **(A)** The 4-h degradation rates of 10 volatile organic compounds at different pH. **(B)** EPR response of the oxidation system at different pH. **(C)** The concentration changing curve of monoaromatics with time at pH 10.0.

The 4-h degradation percentages of the target contaminants at different pH are shown in Figure 1A. The average data and standard deviations of parallel samples are presented. All the control samples showed much lower degradation ratios than test groups. It could be seen that monoaromatics exhibited the highest degradation level compared with chloroalkanes and chloroalkenes, which might be due to the selectivity of ${\text{SO}}_{4}\cdot^{-}$. It is generally accepted that ${\text{SO}}_{4}\cdot^{-}$ reacts through three main pathways, including hydrogen atom abstraction, ${\text{SO}}_{4}\cdot^{-}$ addition on unsaturated bonds, and electron transfer, among which electron transfer is of the most importance. It has been revealed that aromatic compounds were easily oxidized by ${\text{SO}}_{4}\cdot^{-}$, and the presence of chlorines was expected to slow oxidation rate (Ahmed et al., 2012; Xiao et al., 2018). Chlorinated saturated hydrocarbon was resistant to oxidation according to previous studies (Huang et al., 2005; Tsitonaki et al., 2010; Zhu et al., 2016). As for all the monoaromatics and most of the chlorohydrocarbon, the highest degradation ratio was obtained at pH 10.0. For example, chlorobenzene was degraded by about 96% at pH 10.0, while the degradation ratios were 64, 53, and 45% at pH 3.0, 7.0, and 12.0, respectively. In order to explore the reason, the EPR responses of the oxidation system at different pH were determined, and the results are shown in Figure 1B. The strongest signal of ${\text{SO}}_{4}\cdot^{-}$ was observed at pH 10.0. The reason might be that on the one hand, the base acted as the activator of persulfate to produce ${\text{SO}}_{4}\cdot^{-}$, as shown Equations (1–3) (Furman et al., 2010). On the other hand, quinone reacted with HO_2_ to generate semiquinone radical, which accelerated the production of ${\text{SO}}_{4}\cdot^{-}$, as presented in Equations (4, 5). In addition, anionic form of phenols at alkaline pH was the activating species in persulfate systems (Ocampo, 2009; Ahmad et al., 2013), as shown in Equations (6, 7). But as the alkalinity became stronger, ${\text{SO}}_{4}\cdot^{-}$ might react with the reductive radicals, thus weakened the oxidation effect, as shown in Equation (8). An additional experiment showed that carbon tetrachloride was degraded by 10.62 ± 1.03% during 4 h at pH 10.0 while the degradation ratio was negligible in the control without quinone. It verified the occurrence of reductive radicals in this oxidation system.

(1) $$\begin{array}{c} S_{2}O_{8}^{2-}+2OH^{-}\to HO_{2}^{-}+SO_{4}^{2-}+H^{+} \end{array}$$

(2) $$\begin{array}{c} HO_{2}^{-}+S_{2}O_{8}^{2-}\to SO_{4}\cdot^{-}+SO_{4}^{2-}+HO_{2}. \end{array}$$

(3) $$\begin{array}{c} SO_{4}\cdot^{-}+OH^{-}\to SO_{4}^{2-}+OH\cdot \end{array}$$

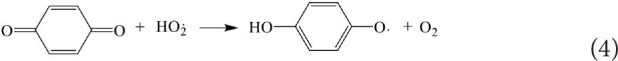

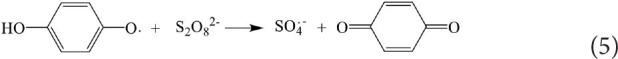

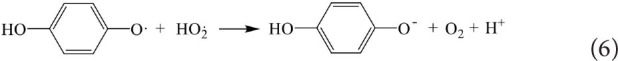

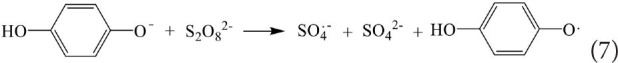

(8) $$\begin{array}{c} HO_{2}^{-}+SO_{4}\cdot^{-}\to SO_{4}^{2-}+HO_{2}\cdot \end{array}$$

### Effect of Initial Quinone Concentration

**Figure 2:**
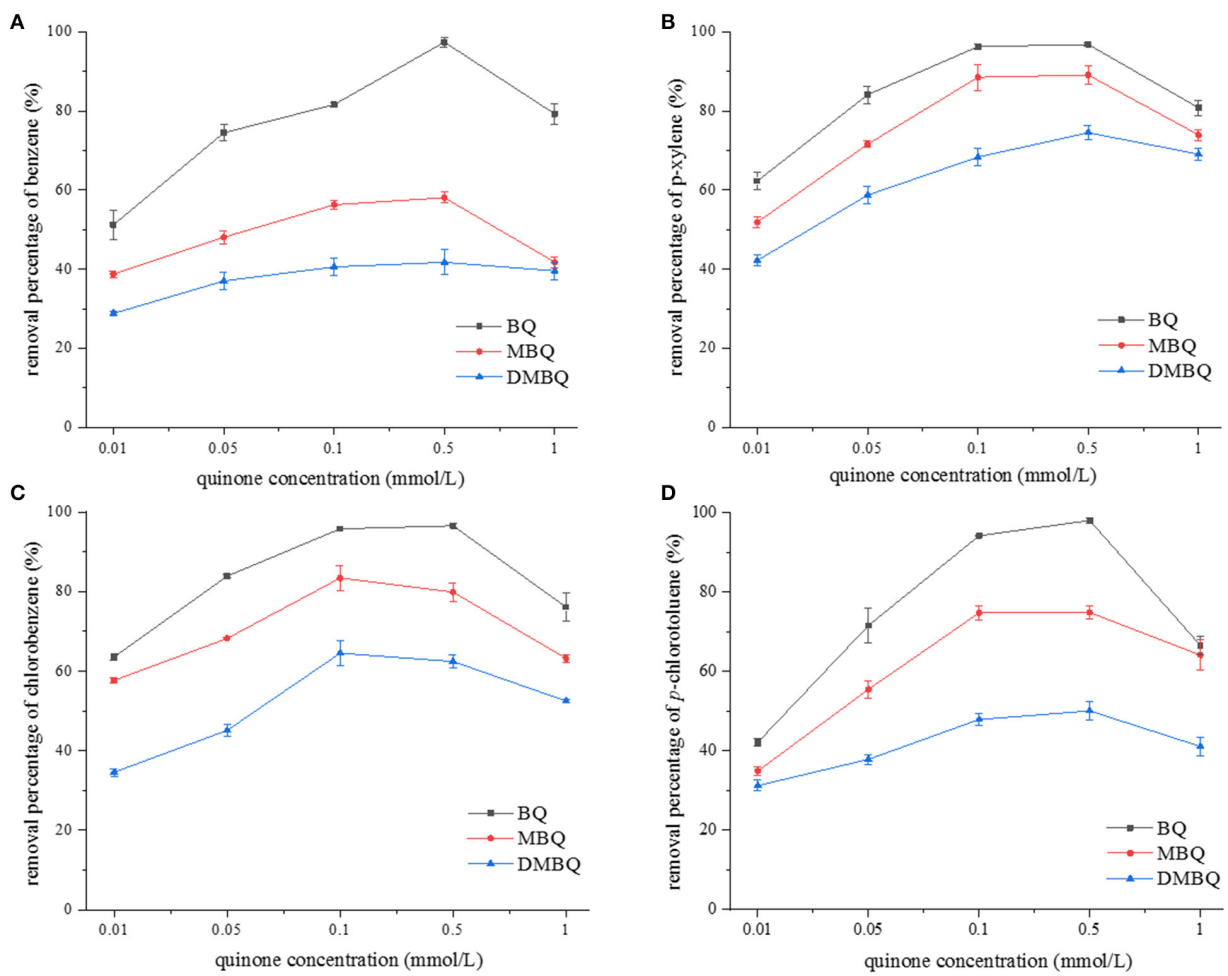
The effect of quinone concentration on 4-h degradation rate of **(A)** benzene, **(B)** p-xylene, **(C)** chlorobenzene, and **(D)** p-chlorotoluene.

In this work, we compared the effect of BQ, MBQ, and DMBQ on the 4-h degradation rate of representative monoaromatics (benzene, *p*-xylene, chlorobenzene, and *p*-chlorotoluene) at pH 10.0. The initial quinone concentrations were set as 0.01, 0.05, 0.1, 0.5, and 1.0 mM, respectively, while the concentration of Na_2_S_2_O_8_ and monoaromatics were the same as above experiments. The results are shown in Figure 2, which indicated that BQ activated persulfate showed the strongest oxidation effect, followed by MBQ and DMBQ. The degradation rate of the monoaromatics increased with the quinone concentration, and reached a plateau at 0.1 or 0.5 mM of quinone, and then dropped gradually. When the amount of quinone was further increased, MBQ and DMBQ were difficult to be dissolved. The results were a little different from those observed by Fang et al. (2013). The amount of oxidative radicals reached the maximum when MBQ increased to 0.2 mM, and then decreased with a higher MBQ concentration in their observation. The difference might be attributed to the fact that the effective quinone concentration range was enlarged in alkaline condition.

### Calculation of Kinetic Constant

As shown in Figure 1C, a rapid decrease of the monoaromatic concentration was observed in the first 2 h, followed by a slow reduction process, which might be related with the decreased amount of ${\text{SO}}_{4}\cdot^{-}$ (Fang et al., 2013).

The degradation kinetics of 20 kinds of monoaromatics (100 μg/L) by 1,4-benzoquinone (0.1 mM) activated persulfate at pH 10.0 was investigated. The samples were collected at 0, 2, 5, 10, 15, and 20 min, respectively, and it was found that each of the oxidation process followed pseudo-first-order kinetics in the first 20 min. The pseudo-first-order kinetic model is shown in Equation (9), in which *k* is the pseudo-first-order reaction rate constant, *t* is the reactive time, and *C*_t_ is the concentration at *t* time (Shi et al., 2017; Xu et al., 2018).

(9) $$\begin{array}{c} dC_{t}/dt=-kC \end{array}$$

According to the pseudo-first-order kinetic model, the reaction rate constant and *R*^2^ of 20 kinds of monoaromatics were calculated and shown in Table 3. The *R*^2^ showed that the values of *k* all fitted well with the pseudo-first-order kinetic model.

**Table 3:** The degradation rate constant (*k*) and *R*^2^ of 20 kinds of monoaromatics.

**No**.	**Compound**	***k***	**–log*k***	***R*^**2**^**
1	benzene	0.0367	1.435	0.9551
2	toluene	0.0832	1.080	0.9366
3	ethylbenzene	0.0889	1.051	0.9735
4^*^	n-propylbenzene	0.1099	0.959	0.9931
5	isopropylbenzene	0.0841	1.075	0.9911
6	n-butylbenzene	0.0728	1.138	0.8979
7	tert-butylbenzene	0.0552	1.258	0.9866
8^*^	para-xylene	0.0778	1.109	0.9854
9	ortho-xylene	0.1102	0.958	0.9984
10	1,3,5-trimethylbenzene	0.1161	0.935	0.9999
11	1,2,4-trimethylbenzene	0.0929	1.032	0.9737
12^*^	para-isopropyltoluene	0.1017	0.993	0.8631
13	chlorobenzene	0.0240	1.620	0.9378
14	bromobenzene	0.0409	1.388	0.9750
15	para-chlorotoluene	0.0719	1.143	0.9202
16^*^	ortho-chlorotoluene	0.0752	1.124	0.9481
17	1,4-dichlorobenzene	0.0171	1.767	0.9211
18	1,2-dichlorobenzene	0.0275	1.561	0.9673
19	1,2,4-trichlorobenzene	0.0371	1.431	0.9972
20^*^	styrene	0.0893	1.049	0.9945

* *Test set chemicals*.

### Construction of QSAR Model

The QSAR model was established by the training set data, as shown in Equation (10). The model revealed that the reaction rate constant had a significant correlation with $q_{\text{C}}^{-}$. The *R*^2^ of the model was >0.80, and the *SEE* and high *F* values was low, indicating a good fit of the model. The $Q_{\text{LOO}}^{2}$ and *RMSE*_CV_ values meant that the internal predictability of the model was satisfactory. The $R_{\text{pred}}^{2}$ and *RMSEp*-values suggested that the established models had a good external predictive ability.

(10) $$\begin{array}{c} -logk=1.735q_{C}^{-}+1.687 \end{array}$$

*R*^2^ = 0.825, *SEE* = 0.105, *F* = 61.215, $Q_{\text{LOO}}^{2}$ = 0.766, *RMSE*_CV_ = 0.121, $R_{\text{pred}}^{2}$ = 0.636, *RMSEp* = 0.133.

The $q_{\text{C}}^{-}$ is an electrostatic descriptor which reflects the non-uniformity of the charge distribution of the molecular, and it has been found to be correlated to the kinetic constant in several previous researches (Zhu et al., 2014, 2016; Huang et al., 2015). A stronger negative net charge of carbon atoms indicated the ease of some valence-bond breakage of organic molecules, so $q_{\text{C}}^{-}$ would have a negative influence on degradation rate. A molecule with a smaller $q_{\text{C}}^{-}$ often gets a higher reaction rate.

**Figure 3:**
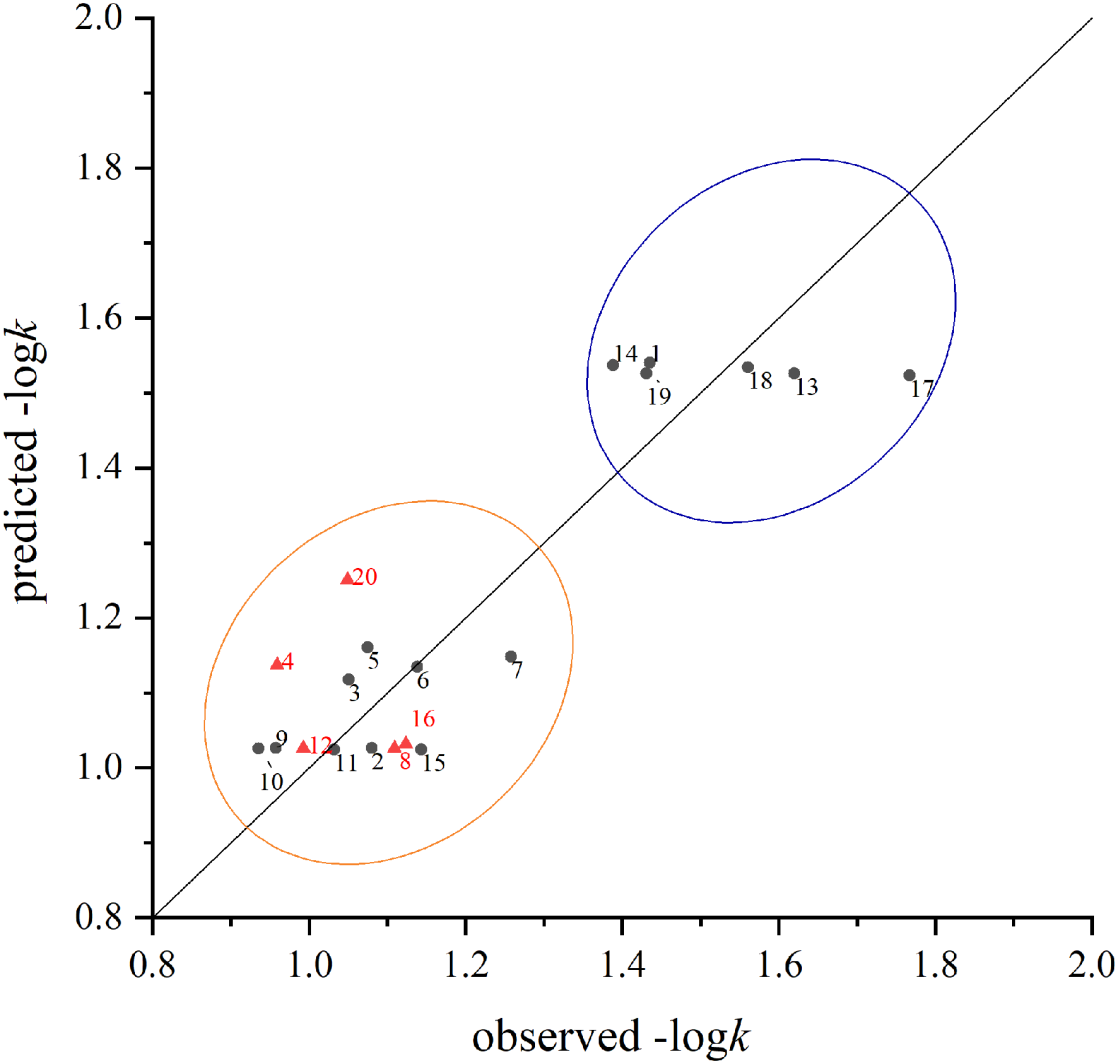
The comparison between the predicted values and measured values of the training set (black circle) and test set (red triangle) in the model. The number beside the point responses to the sequence number listed in Table 2.

The scatter plots of experimental vs. predicted values of –log*k* for the training and test sets are shown in Figure 3. In general, the values predicted by QSAR model were close to the experimental values. The relative residuals between the observed and predicted values were all below 20%. Figure 3 also showed that the chemicals were generally divided into two groups according to their degradation rates. Chemicals with alkyl groups (especially methyl groups) seemed to have a higher degradation rate, while those with halogen substitution usually suffered a low degradation rate.

## Conclusion

This manuscript revealed the influencing factors of monoaromatic oxidation process by quinone-activated persulfate in aqueous solution. The pH, quinone species and concentration all affected the degradation efficiency. Monoaromatics were more easily oxidized in alkaline condition. The reasons were speculated as that base acted as the activator of persulfate to produce ${\text{SO}}_{4}\cdot^{-}$, and the reductive radicals accelerated the semiquinone/quinone cycle, facilitating the production of ${\text{SO}}_{4}\cdot^{-}$. Anionic form of phenols at alkaline pH was also the activating species in persulfate systems. However, excessive reductive radicals in strong alkaline environment inhibited the reaction. The degradation rate increased with quinone concentration, but excess quinone would compete for the oxidant and suppress the degradation of target contaminant. The initial degradation rates of monoaromatics fitted well with the pseudo-first-order equation, and a QSAR model relating the reaction rate with the chemical structure showed good correlation and predictability. The model revealed that the degradation efficiencies of monoaromatics were negatively correlated with $q_{\text{C}}^{-}$. A smaller $q_{\text{C}}^{-}$ meant the non-uniform charge distribution of the molecular, resulting in easy breakage of the bond. This finding would contribute to further explore the effective activating method of persulfate in alkaline environment.

## Data Availability Statement

The original contributions presented in the study are included in the article/supplementary material, further inquiries can be directed to the corresponding author.

## Author Contributions

JS designed the experiments and wrote the manuscript. CJ, DP, and YZ performed the experiments. LW analyzed the data. TL and XZ reviewed this paper and gave some advices. All authors contributed to the article and approved the submitted version.

## Conflict of Interest

The authors declare that the research was conducted in the absence of any commercial or financial relationships that could be construed as a potential conflict of interest.

## Publisher's Note

All claims expressed in this article are solely those of the authors and do not necessarily represent those of their affiliated organizations, or those of the publisher, the editors and the reviewers. Any product that may be evaluated in this article, or claim that may be made by its manufacturer, is not guaranteed or endorsed by the publisher.
